# Comparative Impact of Clear Aligners and Traditional Fixed Appliances on Oral Health-Related Quality of Life: A Systematic Review and Meta-Analysis

**DOI:** 10.3390/medicina62061090

**Published:** 2026-06-04

**Authors:** Elisabetta Lalli, Elena Caramaschi, Agnese Nieri, Sara Vitale, Maurizio Ledda, Giulia Porcu, Pier Luigi Delogu, Alessio Verdecchia, Enrico Spinas

**Affiliations:** 1 Department of Surgical Sciences, Postgraduate School in Orthodontics, University of Cagliari, 09124 Cagliari, Italy; dottoressa.lalli@gmail.com (E.L.); caramaschi.elena@gmail.com (E.C.); agnese.nieri@gmail.com (A.N.); vitale.sara94@gmail.com (S.V.); maurizioledda93@gmail.com (M.L.); giuli.porcu98@gmail.com (G.P.); pierluigidelogu62@gmail.com (P.L.D.); 2 Orthodontics Division, Instituto Asturiano de Odontologia, Universidad de Oviedo, 33006 Oviedo, Spain

**Keywords:** clear aligners, fixed appliances, oral health-related quality of life, OHIP-14, orthodontics, patient-centered care

## Abstract

*Background and Objectives*: The impact of orthodontic appliances on oral health-related quality of life (OHRQoL) has gained increasing attention, particularly in relation to patient-centered outcomes. Clear aligners (CAs) are often perceived as more comfortable than fixed appliances (FAs), although evidence remains limited and heterogeneous. This systematic review and meta-analysis aimed to compare the effects of CAs and FAs on OHRQoL in patients with Angle class I malocclusion and to further evaluate the reliability of the findings using trial sequential analysis (TSA). *Materials and Methods*: This review followed PRISMA guidelines and was registered in PROSPERO (CRD420251051003). A comprehensive search of PubMed, Scopus, Web of Science, Embase, and Cochrane CENTRAL was conducted. Only randomized controlled trials comparing CAs and FAs and reporting OHRQoL outcomes (OHIP-14) were included. Random-effects meta-analyses were performed on early changes in OHRQoL. TSA was applied to control for type I and II errors and to evaluate the sufficiency of the cumulative evidence. *Results*: Two randomized controlled trials (n = 74) were included in the quantitative synthesis. Both treatments were associated with a transient deterioration in OHRQoL after appliance placement. Meta-analysis showed statistically significant improvements favoring CAs in psychological discomfort (*p* = 0.007) and psychological disability (*p* < 0.001), while no significant differences were observed for other domains or for overall OHRQoL. Exploratory TSA suggested an early signal in favor of psychological outcomes, with the cumulative Z-curve crossing the monitoring boundary; however, given the very small number of included trials, these findings should be regarded as preliminary and hypothesis-generating rather than confirmatory. In contrast, for most other outcomes, the required information size was not reached and results remained inconclusive. For handicap, TSA suggested that a clinically relevant difference is unlikely. *Conclusions*: Clear aligners may provide a modest short-term advantage over fixed appliances in psychological aspects of OHRQoL, while no consistent differences are observed in physical or overall domains, based on very limited evidence. TSA suggests a preliminary signal in psychological outcomes but highlights insufficient evidence for most other domains. These results should be interpreted with caution, and further high-quality randomized trials are needed.

## 1. Introduction

Contemporary orthodontics is increasingly characterized by a balance between the patient’s functional, esthetic and psychosocial needs. While the traditional goal of achieving a functionally stable and esthetically harmonious occlusion remains central, growing attention is now devoted to the patient’s overall well-being throughout the course of therapy. In this context, the assessment of oral health-related quality of life (OHRQoL) has emerged as a key parameter in defining treatment success, particularly in adult patients [1].

Over the past two decades, the introduction of clear orthodontic aligners has profoundly transformed the therapeutic landscape. Although the concept of removable appliances dates back to the mid-20th century [2], it was only from the late 1990s onward, with the introduction of the Invisalign^®^ system, that aligners gained widespread popularity [3]. Advances in digital technologies, computer-aided design, and 3D printing have enabled virtual planning and the sequential production of customized aligners, significantly enhancing their clinical effectiveness and patient acceptability [4,5]. Interest in this approach has increased rapidly, particularly among adult patients engaged in professional and social activities [6,7,8].

Despite their popularity, concerns have been raised regarding the clinical effectiveness of aligners compared with fixed orthodontic appliances, often preferred in more complex orthodontic cases, as they allow greater control of three-dimensional tooth movements [9,10,11]. Fixed appliances are generally associated with greater control of three-dimensional tooth movements, including extraction cases, complex rotations, and root torque management [12]. However, some studies suggest that, in selected patients with mild to moderate malocclusions, clear aligners may achieve acceptable clinical out-comes comparable to those obtained with fixed appliances [5,13].

At the same time, increasing attention has been devoted to patient-reported outcomes. OHRQoL is a multidimensional construct encompassing functional, psychological, and social aspects, reflecting the impact of orthodontic treatment on daily life [14,15]. Several studies indicate that aligners are associated with reduced pain, fewer interferences with speech and mastication, lower esthetic impact, and greater social acceptance, particularly during the initial phases of treatment [8,9,15,16,17]. These factors may be clinically relevant when treatment options achieve similar final occlusal results [18,19], as the patient’s subjective experience can influence adherence, satisfaction, and perception of outcomes. Despite the growing body of literature on patient-reported outcomes in orthodontics, the existing evidence base comparing clear aligners and fixed appliances in terms of OHRQoL remains methodologically heterogeneous, with most prior reviews qualitatively synthesizing observational or mixed designs without addressing the sufficiency of the cumulative evidence. To our knowledge, despite the limited number of available randomized trials, no previous quantitative synthesis has applied trial sequential analysis in this field, which makes TSA particularly informative to assess whether the available data are sufficient to draw reliable conclusions or whether further studies are required.

The present systematic review and meta-analysis aim to compare fixed orthodontic appliances and clear aligners, in terms of OHRQoL, under the assumption of comparable clinical outcomes, as reported in the included studies. To minimize clinical heterogeneity that could confound patient-reported outcomes, this review restricts inclusion to patients with Angle class I malocclusion, allowing a more reliable comparison of OHRQoL between treatment modalities. By synthesizing the available evidence, including quantitative analysis where appropriate, this review seeks to support evidence-based, patient-centered decision-making in modern orthodontic practice.

## 2. Materials and Methods

### 2.1. Protocol and Registration

The present systematic review was performed in accordance with the PRISMA statement of Preferred Reporting Items for Systematic Reviews and Meta-Analyses (PRISMA) [20]. The protocol was registered in PROSPERO, the international prospective register of systematic reviews, under the number CRD420251051003.

To define the parameters of the search strategy, we formulated the following target question: “(P) In patients undergoing orthodontic treatment, (I) how do clear aligners compare (C) to traditional fixed appliances (O) in terms of oral health-related quality of life?”

Studies were considered eligible if they met the following PICO criteria:

**P**: Adult patients in permanent dentition with Angle class I malocclusion, requiring non-extraction orthodontic treatment without orthognathic surgery or temporary anchorage devices.

**I**: Studies concerning orthodontic treatment with clear aligners.

**C**: Studies comparing orthodontic treatment with traditional orthodontic fixed appliances.

**O**: Studies that directly report OHRQoL, including pain, discomfort, functional limitations, speech limitations, psychological impact, and social well-being.

### 2.2. Information Sources and Search Strategy

A structured and systematic search of the literature was carried out across multiple electronic databases, including PubMed, Scopus, Web of Science, Embase, and the Cochrane Central Register of Controlled Trials (CENTRAL). Searches were performed up to 15 March 2026, without applying any restrictions regarding publication date. English was the selected language.

For all databases, an identical search framework was adopted, integrating **MeSH (medical subject heading) terms** with free-text keywords and appropriate Boolean operators. Detailed search strings specific to each database are provided in Table 1.

### 2.3. Selection Process and Eligibility Criteria

Four authors (A.N., S.V., E.L., and E.C.) independently conducted the literature search and subsequently screened the retrieved records. To assess the level of agreement among reviewers, Cohen’s kappa coefficient [21] was calculated. Values between 0.61 and 0.80 were considered indicative of substantial agreement. Disagreements among reviewers were discussed collectively until a consensus was reached. In cases where consensus could not be achieved, a fifth reviewer (A.V.) was consulted to make the final decision. A substantial level of agreement was observed (Cohen’s kappa: 0.66). Initial screening was performed based on titles and abstracts, followed by full-text assessment of potentially eligible studies according to the predefined inclusion and exclusion criteria.

The inclusion criteria were: (1) randomized clinical trials (RCTs), included to minimize clinical heterogeneity and improve the reliability of patient-reported outcomes; (2) patients with permanent dentition; (3) patients requiring orthodontic treatment with clear aligners or traditional orthodontic fixed appliances; (4) studies on patients with class I malocclusion to reduce sample heterogeneity; and (5) articles directly reporting OHRQoL, including pain, discomfort, functional limitations, speech limitations, psychological impact, and social well-being.

The exclusion criteria were: (1) studies involving patients in mixed and deciduous dentition; (2) studies involving patients with class II and class III malocclusions; (3) studies including orthodontic appliances other than clear aligners or conventional fixed appliances (e.g., lingual appliances, low-friction fixed appliances, ceramic fixed appliances); (4) studies involving patients with systemic diseases or disorders affecting oral health; (5) studies in which orthodontic treatment required extraction therapy; (6) studies involving orthognathic surgery; (7) studies including the use of temporary anchorage devices (TADs); and (8) non-randomized studies, including cohort, cross-sectional, prospective or retrospective studies, as well as reviews, case reports, case series, editorials, letters, commentaries, conference abstracts, gray literature, and non-comparative studies.

Only studies directly comparing clear aligners and conventional fixed appliances and reporting quantitative OHIP-14 data suitable for longitudinal comparison were considered eligible for meta-analysis.

### 2.4. Data Collection

The four reviewers extracted and elaborated the data of interest according to the Cochrane Consumers and Communication Review Group data extraction model.

The following data were extracted:

• Authors and years of publication;
• Study design;
• Sample size of study group and of control group;
• Gender of participants;
• Age;
• Type of technique used in fixed appliance treatment;
• Aligners’ brand;
• Orthodontic auxiliaries;
• Number of attended visits;
• Overall duration of orthodontic treatments;
• Patient-applied auxiliaries and patients’ compliance;
• OHRQoL outcomes;
• Main results.

In addition, for meta-analytical purposes, mean overall OHIP-14 scores and individual domain scores (functional limitation, physical pain, psychological discomfort, physical disability, psychological disability, social disability, and handicap) at baseline and at the earliest comparable follow-up were extracted, together with measures of dispersion (standard deviation, interquartile range, or range) and sample size for each treatment group at each time point. The primary outcomes registered were the overall OHRQoL score and the corresponding OHIP-14 domain scores; no secondary outcomes were evaluated in this review.

### 2.5. Quality Assessment

All studies included in the review were subjected to a methodological quality appraisal. The risk of bias in the randomized clinical trials was independently evaluated by four authors (E.L., E.C., S.V., and A.N.) using the Cochrane Collaboration risk of bias tool (RoB 2) [22]. This instrument examines potential bias across five specific domains: the randomization procedure, deviations from the intended interventions, and completeness of outcome data, outcome measurement, and selective reporting of results. Based on this assessment, each study was ultimately classified as presenting a low risk of bias, some concerns, or a high risk of bias. In case of disagreement, consensus was reached by majority decision. In the event of a tie, a fifth reviewer (A.V.) was consulted. To assess the certainty of the evidence and the strength of recommendations, the Grading of Recommendations, Assessment, Development, and Evaluation (GRADE) approach [23] was applied.

### 2.6. Statistical Analysis

When sufficient comparable data were available, a meta-analysis was performed to evaluate the effect of orthodontic appliance type on changes in oral health-related quality of life.

Quantitative synthesis focused on the early phase of treatment and was based on the change in OHIP-14 scores from baseline (T0) to approximately one month of follow-up (T1). For each study, the mean change was calculated as the difference between follow-up and baseline values, with negative values indicating improvement in quality of life and positive values indicating deterioration.

When standard deviations of change were not directly reported, they were estimated using established statistical methods. In studies reporting medians and interquartile ranges, means and standard deviations were estimated using the method described by Wan et al. [24]. To compute the standard deviation of change scores, an imputed correlation coefficient of r = 0.488 between baseline and follow-up measurements was applied in accordance with the Cochrane Handbook recommendations. This value was estimated using Spearman’s correlation derived from the available OHIP-14 domain score means across studies.

Meta-analysis was conducted by calculating weighted mean differences (WMDs) between CA and FA groups using a random-effects model, accounting for expected clinical and methodological heterogeneity. Results were reported with 95% confidence intervals (CIs), Z statistics, and corresponding *p*-values.

Statistical heterogeneity was assessed using Cochran’s Q test and quantified with the I^2^ statistic, with values of approximately 25%, 50%, and 75% indicating low, moderate, and high heterogeneity, respectively. Assessment of publication bias through funnel plot analysis or Egger’s test was not performed due to the limited number of included studies. Statistical significance was set at α = 0.05. All analyses were performed using the metafor package in R (version 4.3.1; R Foundation for Statistical Computing, Vienna, Austria).

#### Trial Sequential Analysis (TSA) on Meta-Analysis

TSA was performed to evaluate the reliability of the meta-analytic findings and to control for type I and type II errors. The required information size (RIS) and O’Brien–Fleming monitoring boundaries were calculated assuming a type I error of 5% and a type II error of 20% (power = 80%). The anticipated effect size was defined as a mean difference of 1 in OHIP-14 change scores, based on the results of the random-effects meta-analysis and using the empirical variance. The cumulative Z-curve was examined to assess whether it crossed the trial sequential monitoring boundaries, the futility boundary, or the RIS threshold. All analyses were performed using Trial Sequential Analysis Viewer (TSA Viewer), version 0.9.5.10 Beta (Copenhagen Trial Unit, Copenhagen, Denmark, 2016).

## 3. Results

### 3.1. Study Selection

**Figure 1:**
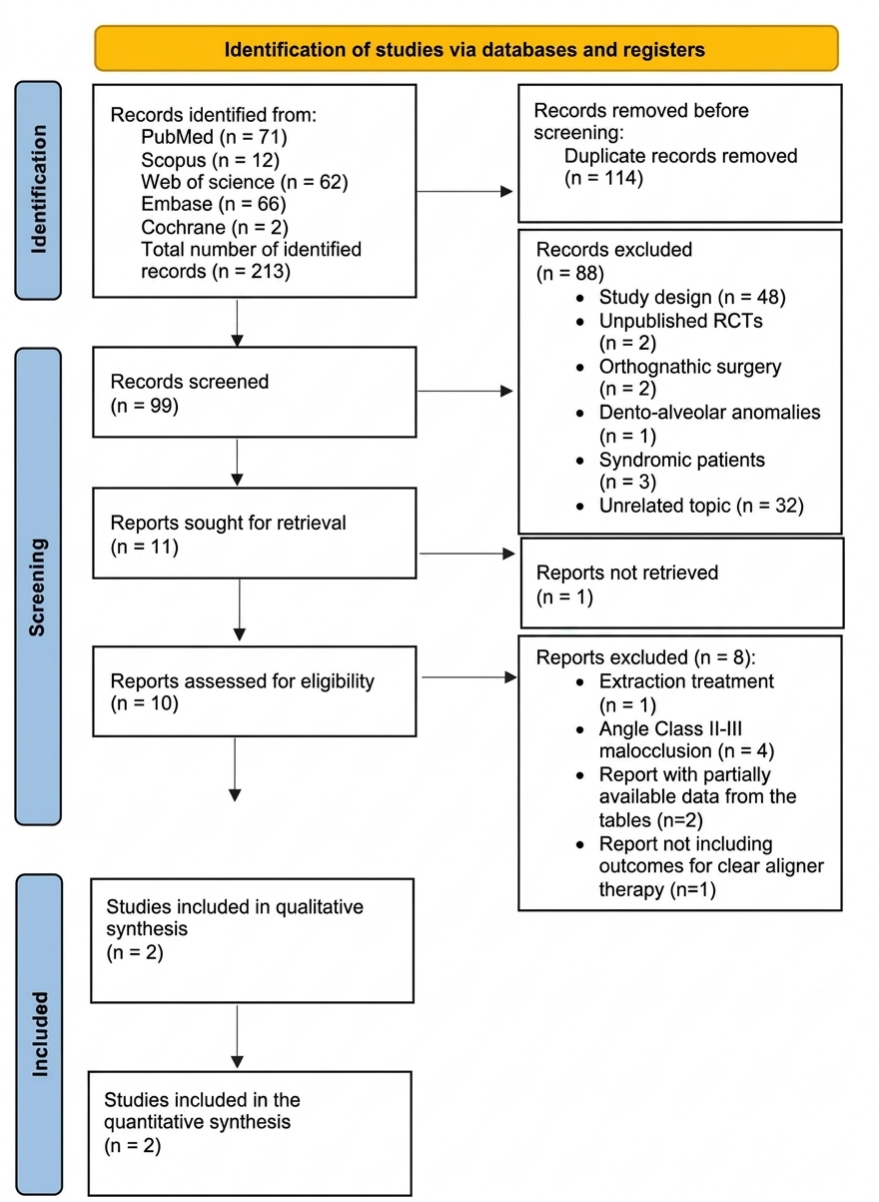
PRISMA flowchart of the performed search.

The flow progression of the records is displayed in Figure 1.

A total number of 213 articles were retrieved across the following databases: Pubmed (71); Web of Science (62); Embase (66); Scopus (12); and Cochrane Library (2). No articles were identified through manual searches or gray literature sources.

A total of 114 articles were removed as duplicates, and 88 were excluded after a review of their title and abstract for the following reasons: inappropriate study design (48), unpublished randomized controlled trials (2), studies focusing on orthognathic surgery (2), dentoalveolar anomalies (1), inclusion of syndromic patients (3), and unrelated topics (32).

Ten articles underwent full-text evaluation for eligibility, where eight were subsequently excluded for the following reasons: extraction treatment (1); Angle class II–III malocclusions (4); reports with partially available Table data (2); and a report not including outcomes for clear aligner therapy (1). Two studies were included in the systematic review.

### 3.2. Sources and Search Strategy

A comprehensive literature search of five databases was undertaken in March 2026, with no temporal restrictions on publication and with inclusion limited to studies published in the English language.

### 3.3. Study Characteristic

Table 2 summarizes the main features of the studies included in the review. Both selected publications date from 2022 to 2025, and the evidence base comprises two randomized controlled trials (RCTs). A standardized Cochrane data extraction template was employed to systematically collect data on study design, participant demographics, baseline malocclusion characteristics, orthodontic treatment modalities, and data collection time points.

#### Sample Characteristic

The studies included in this systematic review feature sample sizes ranging from 36 patients to 38. The pooled sample size across the two included RCTs consisted of a total of 74 patients, which were distributed into two treatment groups, with a relatively balanced distribution between males (34 patients) and females (40 patients) and a mean age ranging from 16.56 to 29.25 years:

Group FA: Total of 37 patients; 18 males − 19 females; mean age = 21.9 ± 3.5 years.

Group CA: Total of 37 patients; 16 males − 21 females; mean age = 22.8 ± 4.5 years.

All patients presented with Angle class I malocclusion, characterized by mild to moderate dental crowding in one or both arches, with reported crowding > 4 mm (mild crowding).

Patients in the FA groups were treated using conventional fixed appliances: Borsato [26] used conventional fixed appliances (0.022 × 0.030-inch slots, 3M Unitek, Monrovia, CA, USA); while Alhafi et al. [25] used MBT prescription.

In the CA groups, clear aligner therapy was provided using InvisalignTM (Align Technology) in Borsato et al.’s [26] study; Alhafi [25] did not disclose the specific type or brand of clear aligners utilized in the publication. The aligners were replaced every 10–14 days, depending on the protocol.

Data collection occurred at multiple time points across studies, generally including:

• T0 = before treatment or after the application of fixed orthodontic attachments in Group FA and the first clear aligner in Group CA.
• T1–T3 = intermediate phases (e.g., 1 week to 12 months).
• T4 = post treatment.

### 3.4. Qualitative Analysis: OHIP-14 Questionnaire Results

Table 3 reports the mean scores for each domain of the OHIP-14 questionnaire across different time points (T0–T4) for both fixed appliance (FA) and clear aligner (CA or MCA) treatment groups.

Overall, both studies demonstrate a transient increase in OHIP-14 scores (indicative of worsened oral health-related quality of life) shortly after appliance placement, followed by a gradual return toward baseline values.

#### 3.4.1. OHIP-14 Domain Scores

As reported by Alhafi et al. [25], at baseline (T0), both groups exhibited similar scores across most domains. Notably, for functional limitation, both FA and MCA started at a mean value of 0.39. In the domain of physical pain, scores peaked at T1 for both groups (FA: 4.17; MCA: 5.89), followed by a progressive decline in subsequent time points, indicating an initial increase in discomfort post-intervention, with a gradual improvement thereafter. A similar trend was observed in the domain of psychological discomfort, where both groups showed a decrease in mean scores from T0 to T4 (FA: from 5.44 to 1.00; MCA: from 5.44 to 1.00), suggesting improved psychological adaptation over time.

In the physical disability domain, scores remained relatively stable from T0 to T2 in the FA group, while the MCA group experienced a more pronounced decline after T1. For psychological disability, both groups exhibited a consistent reduction over time, with more rapid improvement observed in the MCA group.

Social disability and handicap domains displayed relatively low baseline scores and showed a decreasing trend in both groups, indicating a reduction in the perceived social impact and handicap associated with treatment over time.

Overall, both FA and MCA groups demonstrated improvement across all OHIP-14 domains, with a general pattern of initial worsening (particularly evident at T1) followed by progressive recovery. Differences in the magnitude and timing of these changes suggest that the MCA group may experience a more rapid reduction in negative impacts related to oral health quality of life.

As reported by Borsato et al. [26], at baseline (T0), scores were generally low across all domains in both groups, indicating minimal perceived OHRQoL impairment prior to treatment. In the functional limitation domain, both groups showed a modest increase at T1 (FA: 1.74; CA: 0.74), followed by a reduction through T2 and T3, suggesting a temporary increase in functional limitations immediately after treatment initiation.

In the physical pain domain, both groups showed a marked increase at T1 (FA: 4.05; CA: 2.25), with a subsequent decline at T2. However, a slight increase was noted again at T3 in the FA group (2.42), while the CA group continued to improve (1.60), indicating a more consistent pain reduction in the CA group over time.

The psychological discomfort scores peaked at T1 in both groups (FA: 3.00; CA: 1.00), followed by a progressive reduction, with the CA group showing a more sustained improvement by T3. Similar trends were observed in the physical disability and psychological disability domains, where FA patients experienced higher levels of impairment at T1 compared to CA patients, followed by a gradual decrease in scores across both groups. In the social disability and handicap domains, both groups exhibited minimal baseline scores, with only modest increases at T1 in the FA group. These domains remained consistently low throughout the study period, particularly in the CA group, suggesting limited social or long-term functional impact.

In the social disability and handicap domains, both groups exhibited minimal baseline scores, with only modest increases at T1 in the FA group. These domains remained consistently low throughout the study period, particularly in the CA group, suggesting limited social or long-term functional impact.

Overall, the data indicate that while both FA and CA treatments initially lead to a temporary deterioration in OHRQoL (most notably in physical pain and psychological discomfort), these effects tend to diminish over time. The CA group generally experienced lower and more stable OHIP-14 scores across all domains, suggesting a less pronounced negative impact on quality of life compared to the FA group.

#### 3.4.2. Overall OHIP-14 Domain Scores

The overall OHIP-14 scores reported in the included studies demonstrate distinct trends in OHRQoL between patients treated with conventional FAs and those treated with CAs, where available.

In the study by Alhafi et al. [25], both FA and CA groups exhibited an initial increase in OHIP-14 scores from baseline T0 to T1, indicating a temporary deterioration in OHRQoL following the start of treatment. The FA group rose from 17.61 to 19.94, while the CA group showed a more pronounced increase from 17.56 to 22.72. However, from T2 onwards, both groups experienced a progressive decline in scores, reflecting gradual adaptation and symptom improvement. By T4, the FA group reached a score of 4.72 and the CA group 4.83.

In Borsato et al.’s [26] study, the FA group showed an increase from 8.16 at T0 to 14.79 at T1, then a gradual decrease through T2 6.84 and T3 8.21. The CA group, starting slightly higher at T0 9.21, also peaked at T1 6.47 and improved over time to 4.89 by T3.

#### 3.4.3. OHIP-14 Score Comparison Between FA and CA Groups

As described in Table 4, across the studies analyzed, the overall OHIP-14 scores varied between patients treated with fixed appliances and those treated with clear aligners, with observable trends at different time points.

The data recorded in the FA and CA groups were comparable, as patients perceived an increase in discomfort, suggesting a similar initial perceived impact on oral health-related quality of life before orthodontic intervention.

At T1, FA patients reported higher OHIP-14 scores compared to CA patients, indicating a greater initial impact on oral health-related quality of life. Specifically, Alhafi et al. [25] reported 19.94 (FA) vs. 22.72 (CA) and Borsato et al. [26] 14.79 (FA) vs. 6.47 (CA).

At T2, the trend continued, as FA scores were consistently higher than CA scores, with values reported by Alhafi et al. [25] (16.22 vs. 14.56) and Borsato et al. [26] (6.84 vs. 5.16).

At T3, both studies showed decreasing scores in both groups, though FA values remained higher: Alhafi et al. [25] (13.06 vs. 12.11) and Borsato et al. [26] (8.21 vs. 4.89).

At T4, only Alhafi et al. [25] reported data for both treatment groups.

According to the studies analyzed, the overall OHIP-14 scores varied between patients treated with fixed appliances and those treated with clear aligners, with observable trends at different time points.

In summary, the overall trend demonstrates a progressive decrease in OHIP-14 scores over time for both treatment groups. However, CA patients consistently exhibited lower scores than those treated with FA (particularly at T1 and T2), suggesting a more favorable short-term impact of clear aligner therapy on perceived oral health-related quality of life.

### 3.5. Risk of Bias and Study Quality

According to the risk of bias 2 (RoB 2) tool [22], the study by Borsato et al. [26] was judged to have a low risk of bias across all domains. Alhafi et al. [25] showed some concerns, which were mainly related to the randomization process and deviations from the intended interventions. No major issues were identified regarding outcome measurement or selective reporting.

**Figure 2:**
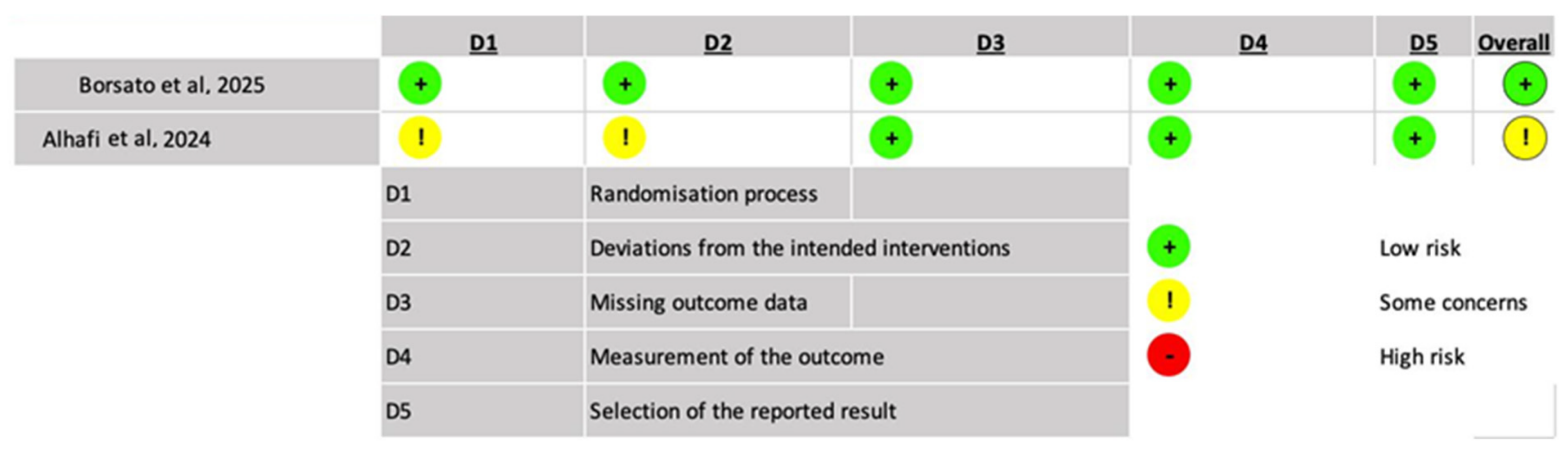
Risk of bias of the included studies, according to the risk of bias (RoB 2) tool [25,26].

Overall, the risk of bias was considered low to moderate across the included studies, as shown in Figure 2.

### 3.6. Quality of Evidence

The quality of evidence for each outcome was assessed using the GRADE approach. The certainty of the evidence was judged as low to moderate for all outcomes, due to the low and some concerns risk of bias and the absence of inconsistency, imprecision, or indirectness. The summary of findings and GRADE ratings are presented in Table 5. The domains analyzed for each article are as follows: functional limitation, physical pain, psychological discomfort, physical disability, and psychological disability.

The quality of evidence for each outcome was assessed using the Grading of Recommendations, Assessment, Development, and Evaluation (GRADE) approach.

The certainty of evidence ranged from low to moderate across the OHIP-14 domains, primarily due to imprecision associated with the small number of included randomized controlled trials, the limited overall sample size, and the absence of pooled estimates, as well as inconsistency related to variability in assessment time points and effect magnitude across studies. No serious concerns were identified regarding indirectness or publication bias. The summary of findings and GRADE ratings are reported in Table 5.

### 3.7. Quantitative Results: Meta-Analysis Outcomes

The two randomized controlled trials included in the systematic review directly comparing clear aligners (CAs) and fixed appliances (FAs) provided quantitative data suitable for meta-analysis. Both studies assessed oral health-related quality of life using the OHIP-14 questionnaire and reported outcomes at baseline (T0) and at an early follow-up approximately one month after treatment initiation. Early changes in OHIP-14 scores (ΔT1–T0) were selected as the primary outcome for quantitative synthesis. Meta-analyses were conducted using a random-effects model to account for expected clinical and methodological heterogeneity between studies. Weighted mean differences (WMDs) between CA and FA groups were calculated for each OHIP-14 domain and for the overall score, together with standard errors (SEs), 95% confidence intervals (CIs), z statistics, and *p*-values. Negative WMD values indicate a greater improvement, or lower deterioration, in oral health-related quality of life favoring clear aligners. Where substantial heterogeneity was observed, pooled estimates should be interpreted with caution. The results of the quantitative synthesis are reported below for each OHIP-14 domain and for the overall score.

#### 3.7.1. Functional Limitation

No statistically significant difference was observed between CAs and FAs (WMD = 0.04; SE = 0.92; 95% CI: −1.76 to 1.84; z = 0.05; *p* = 0.964): both treatments produced a similar effect. Substantial heterogeneity was detected (I^2^ = 93.5%; Cochran’s Q *p* < 0.001), indicating considerable inconsistency across studies.

Trial sequential analysis showed that the cumulative sample size (n = 74) was far below the required information size (RIS = 491), and the Z-curve did not cross monitoring or futility boundaries, indicating that the available evidence is insufficient and the result remains inconclusive.

#### 3.7.2. Physical Pain

No statistically significant difference between treatment modalities was observed for changes in physical pain (WMD = −0.66; SE = 1.31; 95% CI: −3.23 to 1.90; z = −0.51; *p* = 0.611). Heterogeneity was estimated at a high level (I^2^ = 89.7%; Q *p* = 0.001).

TSA indicated that the cumulative sample size (n = 74) was markedly below the RIS (n = 1002), with no crossing of monitoring or futility boundaries, suggesting insufficient evidence and an inconclusive result.

#### 3.7.3. Psychological Discomfort

**Figure 3:**
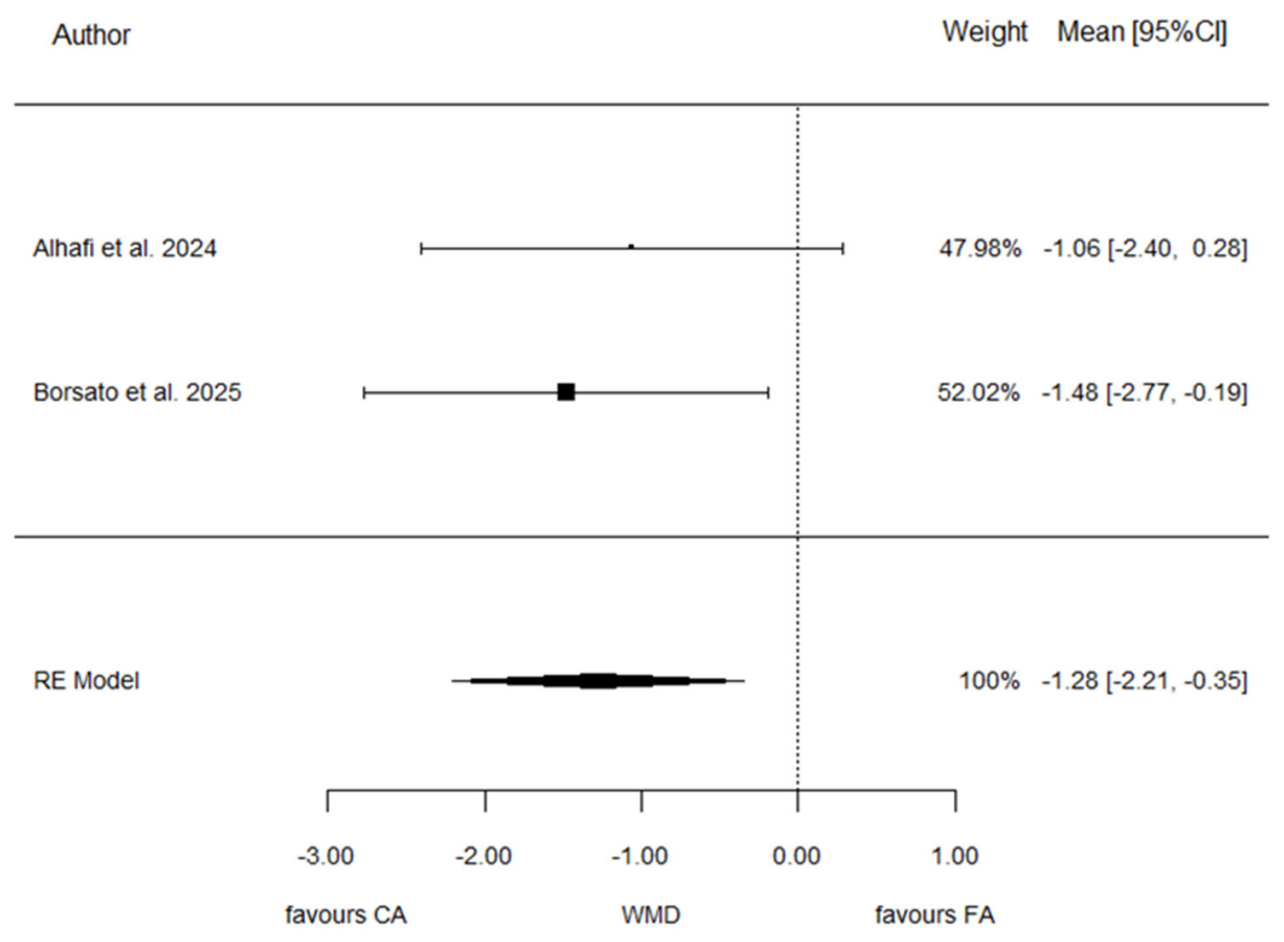
Forest plot of the random-effects meta-analysis comparing the change in OHIP-14 psychological discomfort score from baseline to 1 month between clear aligners and fixed appliances [25,26]. CI, confidence interval; WMD, weighted mean difference.

A statistically significant improvement favoring clear aligners was observed for psychological discomfort (WMD = −1.28; SE = 0.48; 95% CI: −2.21 to −0.35; z = −2.69; *p* = 0.007). No heterogeneity was detected (I^2^ = 0%; Q *p* = 0.658) (Figure 3).

**Figure 4:**
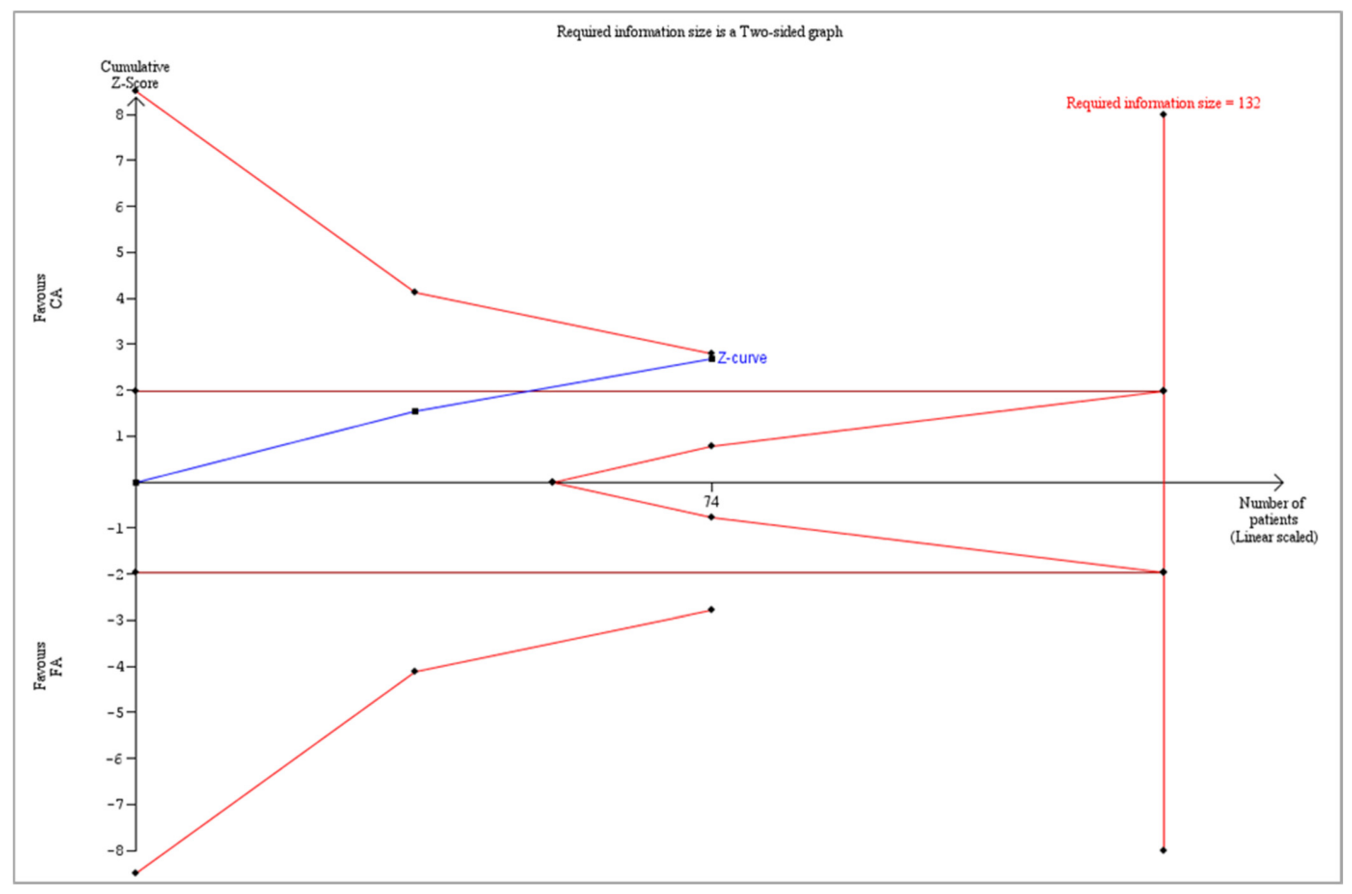
TSA for psychological discomfort.

TSA demonstrated that the cumulative Z-curve crossed the O’Brien–Fleming monitoring boundary despite not reaching the RIS (n = 132), suggesting that the observed effect may reflect an underlying difference. The TSA results are presented in Figure 4.

#### 3.7.4. Physical Disability

The meta-analysis did not demonstrate a statistically significant difference between CAs and FAs for physical disability (WMD = −1.29; SE = 1.07; 95% CI: −3.40 to 0.81; z = −1.21; *p* = 0.228). Substantial heterogeneity was observed (I^2^ = 86.1%; Q *p* = 0.007).

TSA showed that the cumulative sample size (n = 74) did not reach the RIS (n = 677), and the Z-curve did not cross monitoring or futility boundaries, indicating insufficient evidence and inconclusive findings.

#### 3.7.5. Psychological Disability

**Figure 5:**
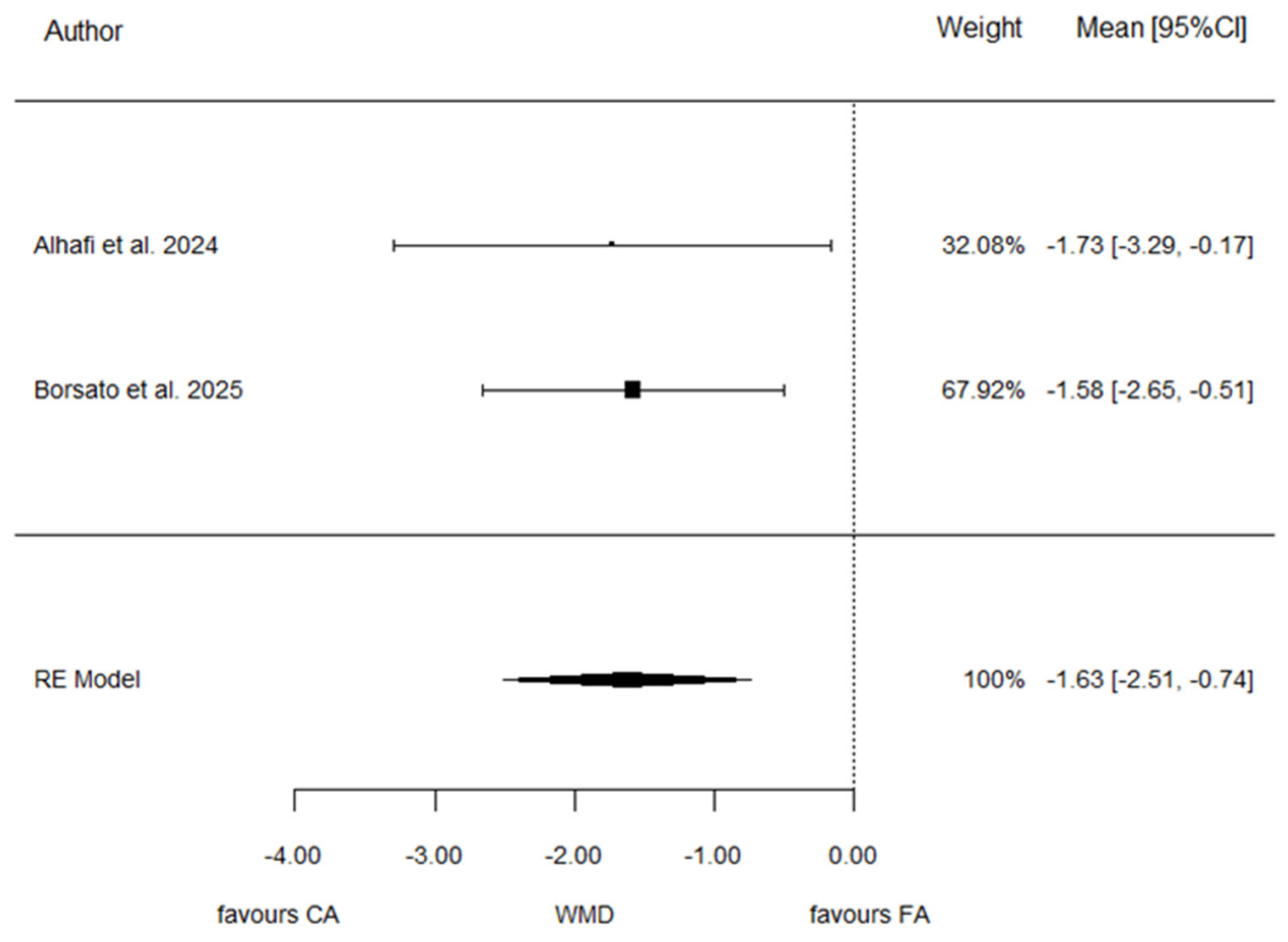
Forest plot of the random-effects meta-analysis comparing the change in OHIP-14 psychological disability score from baseline to 1 month between clear aligners and fixed appliances [25,26]. CI, confidence interval; WMD, weighted mean difference.

Clear aligners were associated with a statistically significant benefit in psychological disability (WMD = −1.63; SE = 0.45; 95% CI: −2.51 to −0.74; z = −3.62; *p* < 0.001). No heterogeneity was observed (I^2^ = 0%; Q *p* = 0.876) (Figure 5).

**Figure 6:**
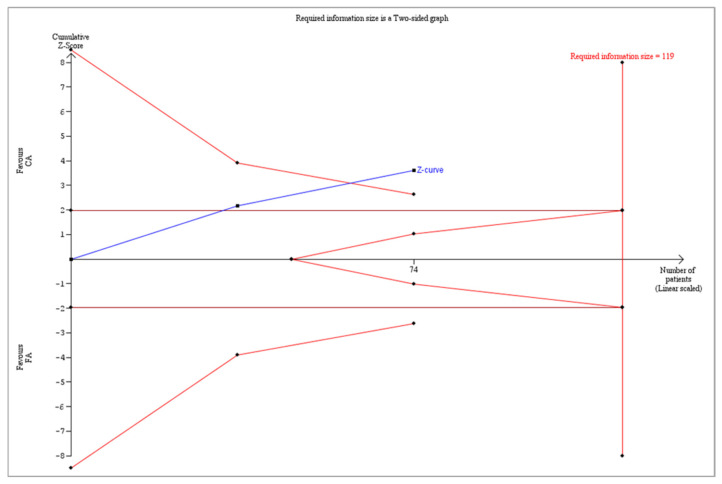
TSA for psychological disability.

TSA indicated that the cumulative Z-curve crossed the O’Brien–Fleming monitoring boundary before reaching the RIS (n = 119), suggesting that the observed effect may reflect an underlying difference (Figure 6).

#### 3.7.6. Social Disability

**Figure 7:**
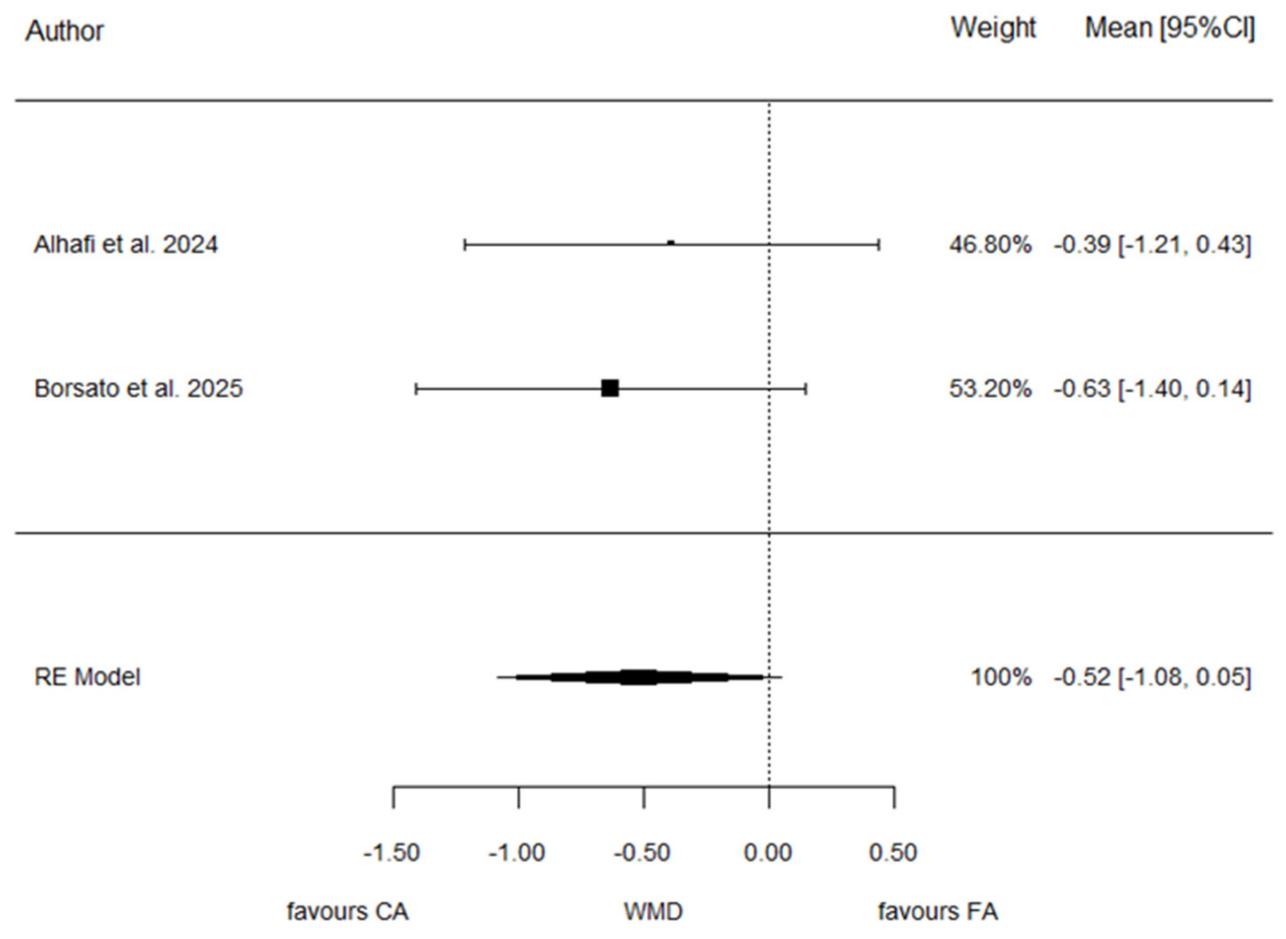
Forest plot of the random-effects meta-analysis comparing the change in OHIP-14 social disability score from baseline to 1 month between clear aligners and fixed appliances [25,26]. CI, confidence interval; WMD, weighted mean difference.

A borderline statistically significant difference favoring clear aligners was identified for social disability (WMD = −0.52; SE = 0.29; 95% CI: −1.08 to 0.05; z = −1.80; *p* = 0.072). No heterogeneity was detected (I^2^ = 0%; Q *p* = 0.677) (Figure 7).

TSA showed that the required information size (RIS = 49) was reached, and the cumulative evidence approached the conventional significance boundary, suggesting a potentially reliable but borderline effect.

#### 3.7.7. Handicap

No statistically significant difference was found between CAs and FAs (WMD = −0.21; SE = 0.37; 95% CI: −0.93 to 0.51; z = −0.58; *p* = 0.561): both treatments produced a similar effect. No heterogeneity was observed (I^2^ = 0%; Q *p* = 0.385).

TSA indicated that although the RIS (n = 79) was not fully reached, the Z-curve crossed the futility boundary, suggesting that a clinically relevant difference between treatments is unlikely.

#### 3.7.8. Overall OHIP-14 Score

**Figure 8:**
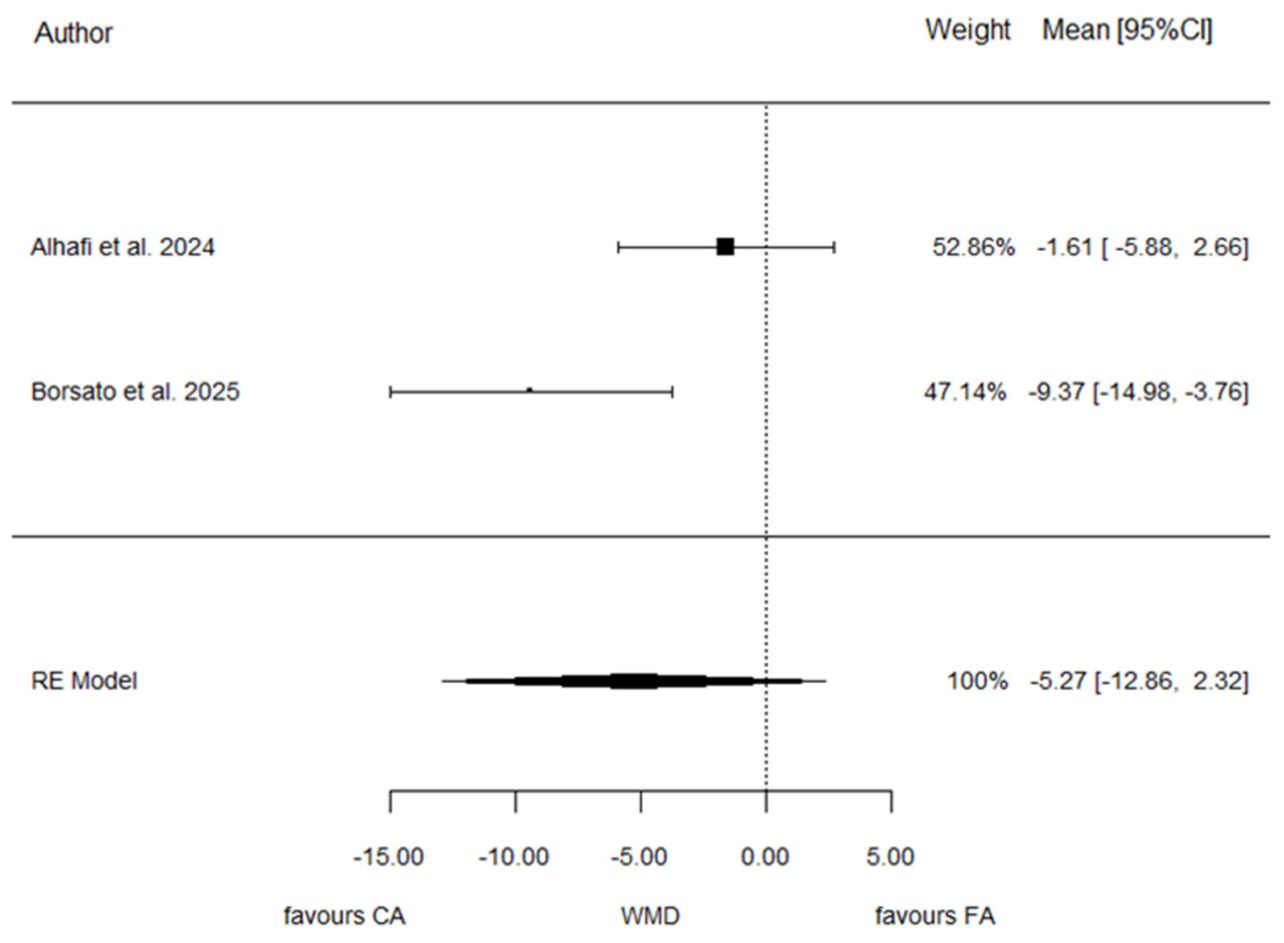
Forest plot of the random-effects meta-analysis comparing the change in overall OHIP-14 score from baseline to 1 month between clear aligners and fixed appliances [25,26]. CI, confidence interval; WMD, weighted mean difference.

The pooled analysis of overall OHIP-14 change scores showed no statistically significant difference between clear aligners and fixed appliances (WMD = −5.27; SE = 3.87; 95% CI: −12.90 to 2.32; z = −1.36; *p* = 0.174). Substantial heterogeneity was detected (I^2^ = 78.5%; Q *p* = 0.031) (Figure 8).

#### 3.7.9. Summary of Meta-Analytic Findings

Overall, statistically significant differences favoring clear aligners were confined to psychological domains, namely psychological discomfort and psychological disability. No significant differences were observed for functional limitation, physical pain, physical disability, handicap, or the overall OHIP-14 score. The limited number of included studies contributed to imprecision of the pooled estimates. Trial sequential analysis supported the findings for psychological domains, while indicating that most other outcomes remain underpowered and require further evidence.

## 4. Discussion

The evaluation of oral health-related quality of life (OHRQoL) has gained increasing attention in orthodontic research, reflecting the growing relevance of patient-reported outcomes alongside traditional clinical parameters. Previous studies have consistently shown that orthodontic treatment is associated with a transient deterioration in OHRQoL, particularly during the early phase following appliance placement, mainly due to pain, functional limitations, and psychological discomfort [1,2,3]. Compared with the previous systematic review by Kaklamanos et al. [1], which qualitatively synthesized prospective studies of mixed design without pooled quantitative estimates, the present meta-analysis advances the existing evidence by providing pooled weighted mean differences for individual OHIP-14 domains, restricting inclusion to RCT in patients with Angle class I malocclusion, and applying TSA to assess the sufficiency of the cumulative evidence. Identifying potential differences between orthodontic appliances during this initial phase is therefore clinically relevant.

Fixed orthodontic appliances remain a widely used treatment modality due to their predictable biomechanical control and established clinical effectiveness [9,10,11,12]. However, their use is frequently associated with discomfort, soft tissue irritation, and esthetic concerns, especially during the first weeks of treatment [12,25,26,27,28]. These factors may temporarily impair OHRQoL and influence patient acceptance of treatment. Clear aligner therapy has been proposed as an alternative approach to improve patient comfort and esthetics while achieving satisfactory clinical outcomes in selected cases [3,4,5,13]. The removable design and reduced visibility of aligners are considered potential advantages in reducing both physical discomfort and psychological burden, particularly during the early adaptation period [6,7,8,15,16,17]. Nevertheless, the extent to which these advantages translate into consistent improvements in OHRQoL remains variable across studies [8,9,18,19,20].

The present meta-analysis quantitatively compared early changes in OHRQoL between clear aligners and fixed appliances in patients with Angle class I malocclusion, using the OHIP-14 questionnaire as a standardized assessment tool [14,15]. To reduce clinical and methodological heterogeneity, only randomized controlled trials directly comparing the two appliance systems were included, and the analysis was restricted to a unified early follow-up time point, approximately one month after treatment initiation. This time point corresponds to the period of greatest reported treatment-related discomfort and was consistently available across the included studies [25,26].

The pooled results confirmed that both treatment modalities are associated with a short-term worsening of OHRQoL after treatment initiation, in line with previous randomized trials and systematic reviews [1,8,18]. However, clear aligners showed significantly greater improvement in psychological domains of the OHIP-14, namely psychological discomfort and psychological disability. These findings suggest that aligner therapy may provide a short-term benefit in psychologically related aspects of OHRQoL, potentially associated with esthetic factors and reduced perceived treatment intrusiveness [6,7,8,15,16,17].

No statistically significant differences were observed between clear aligners and fixed appliances for functional limitation, physical pain, physical disability, handicap, or overall OHIP-14 score. Several of these domains exhibited substantial heterogeneity, indicating variability in patient response. This variability likely reflects the multifactorial nature of OHRQoL, which is influenced by individual pain perception, force application, treatment mechanics, and patient expectations rather than appliance type alone [29,30,31,32,33,34,35]. The substantial heterogeneity observed for several domains (I^2^ > 80%) likely reflects multiple sources of variability that could not be fully controlled despite the strict inclusion criteria. Differences in aligner systems (Invisalign in Borsato et al., undisclosed brand in Alhafi et al.) and in replacement protocols (every 10 vs 10–14 days) may influence the magnitude and timing of OHRQoL changes. Variability in the standardization and timing of OHIP-14 administration, incomplete reporting of patient compliance and adherence, and minor differences in baseline crowding severity may also have contributed to between-study inconsistency. These findings are consistent with previous systematic reviews reporting more favorable short-term patient-reported outcomes with clear aligners in specific domains, while also indicating that differences between appliance systems tend to diminish over time [8,9,15,18,19,20]. The absence of a significant difference in overall OHIP-14 scores supports the interpretation that the observed benefits of aligners are domain specific rather than global.

### Study Limitations

Several limitations of this systematic review and meta-analysis should be clearly acknowledged and must be considered when interpreting the results. First, the limited number of randomized controlled trials included reduces the overall strength of the evidence and limits statistical power. This also precluded a formal assessment of publication bias, which may be relevant in the context of patient-reported outcomes.

Second, some methodological limitations related to data reporting affected the meta-analytic procedures. In one included study, standard deviations for change scores were not directly reported and were therefore estimated using established statistical methods [25]. In addition, the variance of change scores were calculated by imputing a correlation coefficient between baseline and follow-up measurements, as individual patient data were unavailable. Although this approach is commonly used in meta-analyses, the assumed correlation may influence the precision of the pooled estimates. The lack of sensitivity analyses using alternative correlation values represents an additional limitation.

Third, despite efforts to reduce heterogeneity through strict inclusion criteria, residual clinical heterogeneity cannot be excluded. Differences in aligner systems, attachment protocols, treatment staging, and patient compliance—often incompletely reported—may have influenced patient-reported outcomes and contributed to variability among studies [6,8,33]. The restriction to Angle class I malocclusion, although intended to minimize clinical heterogeneity and improve internal validity, inevitably limits the generalizability of the findings to patients with class II or class III malocclusions, in whom appliance-related impact on OHRQoL could differ substantially because of treatment complexity, extended duration, and use of additional auxiliaries [9,10,11]. Moreover, the focus on short-term follow-up does not allow conclusions regarding the persistence or clinical relevance of OHRQoL differences beyond the early phase of treatment.

Although trial sequential analysis suggested more stable evidence for psychological outcomes, most other domains did not reach the required information size, highlighting the limited statistical power and the need for further studies.

From a clinical perspective, these findings carry several patient-relevant implications. The statistically significant benefit of clear aligners in the psychological domains of OHIP-14—namely psychological discomfort (WMD = −1.28) and psychological disability (WMD = −1.63)—although modest in absolute magnitude, may be clinically meaningful for patients in whom esthetic perception and psychosocial comfort drive treatment expectations, particularly among adults engaged in professional and social activities [6,7,8,16]. In the context of shared decision-making, these results support the inclusion of patient-reported outcomes alongside biomechanical considerations when discussing appliance options. Conversely, the absence of consistent differences in functional, physical, and overall OHRQoL domains indicates that clear aligners should not be presented to patients as globally superior, but rather that they offer a domain-specific short-term advantage. Appliance selection should therefore remain individualized and guided by case complexity, biomechanical requirements, clinician experience, and the patient’s own priorities regarding esthetics, comfort, and treatment expectations. From a patient-counseling standpoint, clinicians may use these findings to set realistic expectations, as both appliance systems are associated with a transient early deterioration of OHRQoL, which tends to recover over time, while the psychological adaptation appears smoother with aligners.

Overall, this meta-analysis indicates that clear aligners provide a modest but statistically significant short-term benefit over fixed appliances in specific psychological domains of OHRQoL, while no consistent differences are observed for physical or functional outcomes or for overall OHRQoL. These findings support the use of clear aligners as a patient-centered option in appropriately selected cases, while highlighting the need for cautious interpretation. Further high-quality randomized controlled trials with standardized outcome reporting and longer follow-up are needed to better characterize the temporal evolution of OHRQoL differences between orthodontic appliance systems.

## 5. Conclusions

This meta-analysis compared the early impacts of clear aligners and fixed orthodontic appliances on oral health-related quality of life in patients with Angle class I malocclusion. Both appliance systems were associated with a short-term deterioration in OHRQoL after treatment initiation. Clear aligners showed a modest but statistically significant advantage over fixed appliances in psychological domains of the OHIP-14, while no consistent differences were observed in physical, functional, or overall OHRQoL scores.

These findings indicate that the benefits of clear aligners are limited to specific psychological aspects of quality of life during the early treatment phase and do not translate into a global improvement in OHRQoL. Given the limited number of available randomized trials and the need for variance imputation in one included study, the results should be interpreted with caution. Further well-designed randomized controlled trials with standardized outcome reporting and longer follow-up are required to better define short- and long-term differences in patient-reported outcomes between orthodontic appliance systems.

## Figures and Tables

**Table 1:** Search strategy for each database.

Database	Search Strategy
PubMed	(“clear aligners”[All Fields] OR “Invisalign”[All Fields] OR “thermoplastic aligners”[All Fields] OR “CAT”[All Fields] OR (“fixed orthodontic appliances”[All Fields] OR “brackets”[All Fields] OR “traditional braces”[All Fields])) AND (“oral health-related quality of life”[All Fields] OR “OHRQoL”[All Fields])
Scopus	(TITLE-ABS-KEY (“clear aligners”) OR TITLE-ABS-KEY (“Invisalign”) OR TITLE-ABS-KEY (“thermoplastic aligners”) OR TITLE-ABS-KEY (“CAT”) AND (TITLE-ABS-KEY (“fixed orthodontic appliances”) OR TITLE-ABS-KEY (“brackets”) OR TITLE-ABS-KEY (“traditional braces”))) AND (TITLE-ABS-KEY (“oral health-related quality of life”) OR TITLE-ABS-KEY (“OHRQoL”))
Web of Science	TS=(“clear aligners” OR “Invisalign” OR “thermoplastic aligners” OR “CAT” OR “fixed orthodontic appliances” OR “brackets” OR “traditional braces”) AND TS=(“oral health-related quality of life” OR “OHRQoL”)
Embase	(‘clear aligners’:ti,ab,kw OR ‘invisalign’:ti,ab,kw OR ‘thermoplastic aligners’:ti,ab,kw OR ‘cat’:ti,ab,kw OR ‘fixed orthodontic appliances’:ti,ab,kw OR ‘brackets’:ti,ab,kw OR ‘traditional braces’:ti,ab,kw) AND (‘oral health-related quality of life’:ti,ab,kw OR ‘ohrqol’)
Cochrane Central Register of Controlled Trials	(“clear aligners” OR “Invisalign” OR “thermoplastic aligners” OR “CAT”) AND (“fixed orthodontic appliances” OR “brackets” OR “traditional braces”) AND (“oral health-related quality of life” OR “OHRQoL”)

**Table 2:** The table provides the main features of the two articles included in the systematic review.

Author and Year (Country)	Type of Article	Participants	Mean Age (Year)	Initial Malocclusion	Group FA Orthodontic Treatment	Group CA Orthodontic Treatment	Collection of Data
Alhafi et al., 2024 (Syria) [25]	Single-center, two-arm, parallel-group, single-blinded RCT	36 patients Group FA = 18 (6 M, 12 F) Group CA = 18 (5 M, 13 F)	Group FA = 20.94 ± 2.38 Group CA = 21.89 ± 2.63	Angle class I malocclusion, mild crowding (>4 mm)	Conventional fixed appliance (0.022 × 0.028-inch slots, Master Series^®^, American Orthodontics, Sheboygan, WI, USA); MBT prescription	N/R	T0 = before treatment T1 = 2 weeks T2 = 1 month T3 = 2 months T4 = post treatment
Borsato et al., 2025 (Brazil) [26]	Parallel-group RCT	39 patients Group FA = 19 (12 M, 7 F) Group CA = 20 (12 M, 8 F)	Group FA = 20.91 ± 4.35 Group CA = 23.63 ± 5.62	Angle class I malocclusion, moderate mandibular anterior crowding	Conventional fixed appliance (0.022 × 0.030 inch slots, 3M Unitek, Monrovia, CA, USA)	InvisalignTM (Align Technology, Santa Clara, CA, USA); replaced every 10 days	T0 = before treatment T1 = 1 month T2 = 6 months T3 = 12 months

Glossary of abbreviations: RCT, randomized controlled trial; FA, fixed appliance (conventional braces); CA, clear aligner; MCA, modified clear aligner; M, male; F, female; MBT, McLaughlin–Bennett–Trevisi; N/R, not reported; T0, before treatment; T1, early follow-up (e.g., 1 week, 2 weeks, or 1 month, depending on the study); T2, intermediate follow-up (1–6 months, depending on the study); T3, later follow-up (3–12 months, depending on the study); T4, post treatment. Both studies employed a parallel-group design, comparing fixed orthodontic appliances (Group FA) with clear aligners (Group CA) in patients presenting with Angle class I malocclusion and mild or moderate degrees of dental crowding.

**Table 3:** Mean OHIP-14 domain scores at different time points (T0–T4) for patients treated with fixed appliances (FAs) and clear aligners/modified clear aligners (CAs/MCAs).

Author and Year	OHIP-14 Domains	Group	T0—Mean	T1—Mean	T2—Mean	T3—Mean	T4—Mean
Alhafi et al., 2024 [25]	Functional limitation	FA	0.39	2.33	1.39	0.94	0.72
		MCA	0.39	3.78	2.33	2.17	0.83
	Physical pain	FA	2.33	4.17	3.33	2.72	0.83
		MCA	2.06	5.89	3.67	2.83	0.83
	Psychological discomfort	FA	5.44	4.94	4.28	3.72	1.00
		MCA	5.44	4.78	3.22	2.50	1.00
	Physical disability	FA	1.11	1.94	1.94	1.72	0.72
		MCA	1.11	2.50	1.67	1.44	0.50
	Psychological disability	FA	4.44	4.28	3.67	2.89	0.44
		MCA	4.56	2.94	2.06	1.67	0.56
	Social disability	FA	1.50	1.22	0.89	0.56	0.39
		MCA	1.72	1.50	0.72	0.61	0.39
	Handicap	FA	2.39	1.06	0.72	0.50	0.61
		MCA	2.28	1.33	0.89	0.89	0.72
Borsato et al., 2025 [26]	Functional limitation	FA	0.42	1. 74	0.84	0.95	N/R
		CA	0.32	0.74	0.68	0.68	N/R
	Physical pain	FA	2.05	4.05	2.11	2.42	N/R
		CA	2.26	2.25	2.30	1.60	N/R
	Psychological discomfort	FA	2.47	3.00	1.37	1.95	N/R
		CA	1.95	1.00	0.58	1.05	N/R
	Physical disability	FA	0.63	2.79	1.32	1.11	N/R
		CA	1.42	1.16	0.58	1.05	N/R
	Psychological disability	FA	1.37	1.95	0.89	1.11	N/R
		CA	1.74	0.74	0.53	0.74	N/R
	Social disability	FA	0.63	0.74	0.32	0.53	N/R
		CA	0.89	0.37	0.26	0.42	N/R
	Handicap	FA	0.58	0.53	0.11	0.16	N/R
		CA	0.63	0.16	0.11	0.11	N/R

Glossary of abbreviations: OHIP-14, Oral Health Impact Profile, 14-item questionnaire; FA, fixed appliance (conventional braces); CA, clear aligner; MCA, modified clear aligner (or alternative clear aligner protocol, depending on study); N/R, not reported; T0, before treatment; T1, early follow-up (e.g., 1–2 weeks or 1 month, depending on the study); T2, intermediate follow-up (1–6 months); T3, later follow-up (3–12 months); T4, post treatment.

**Table 4:** Overall OHIP-14 scores at each evaluation time (T0–T4) comparing fixed appliance (FA) and clear aligner (CA) treatment groups.

Author and Year	T0—Overall Score		T1—Overall Score		T2—Overall Score		T3—Overall Score		T4—Overall Score			
	FA	CA	FA	CA	FA	CA	FA	CA	FA	CA	FA	CA
Alhafi et al., 2024 [25]	17.61	17.56	19.94	22.72	16.22	14.56	13.06	12.11	4.72	4.83	N/R	N/R
Borsato et al., 2025 [26]	8.16	9.21	14.79	8.21	8.21	5.16	8.21	4.89	N/R	N/R	N/R	N/R
	9.37	N/R	19.84	6.37	6.37	N/R	6.37	N/R	4.32	N/R	3.37	N/R

Glossary of abbreviations: FA, fixed appliances (conventional braces); CA, clear aligners; N/R, not reported; T0, before treatment; T1, early follow-up (e.g., 1 month, depending on the study); T2, intermediate follow-up (≈6 months, depending on the study); T3, later follow-up (≈ 12 months or end of treatment, depending on the study; T4, post treatment. At baseline (T0).

**Table 5:** Summary of the quality of evidence (GRADE) for the main outcomes evaluated.

Outcome (OHIP-14 Domain)	No. of Studies (Design)	Risk of Bias	Inconsistency	Indirectness	Imprecision	Publication Bias	Overall GRADE
Functional limitation [25,26]	2 (RCTs)	Not serious	Serious	Not serious	Serious	Undetected	Low
Physical pain [25,26]	2 (RCTs)	Not serious	Serious	Not serious	Serious	Undetected	Low
Psychological discomfort [25,26]	2 (RCTs)	Not serious	Not serious	Not serious	Serious	Undetected	Moderate
Physical disability [25,26]	2 (RCTs)	Not serious	Serious	Not serious	Serious	Undetected	Low
Psychological disability [25,26]	2 (RCTs)	Not serious	Serious	Not serious	Serious	Undetected	Low
Social disability [25,26]	2 (RCTs)	Not serious	Not serious	Not serious	Serious	Undetected	Moderate
Handicap [25,26]	2 (RCTs)	Not serious	Not serious	Not serious	Serious	Undetected	Moderate

Glossary of abbreviations: OHIP-14, Oral Health Impact Profile, 14-item questionnaire; RCT, randomized controlled trials.

## Data Availability

The data supporting the findings of the present systematic review are available within the article.
